# Decreased Risk of Low Back Pain During Pregnancy Associated With the Use of Orthopedic Manual Therapy: A Nested Case-Control Study

**DOI:** 10.3389/fmed.2022.887877

**Published:** 2022-06-23

**Authors:** Wei-Chiao Chang, Hanoch Livneh, Chieh-Tsung Yen, Min-Chih Hsieh, Ming-Chi Lu, Wei-Jen Chen, Tzung-Yi Tsai

**Affiliations:** ^1^Department of Chinese Medicine, Dalin Tzuchi Hospital, The Buddhist Tzuchi Medical Foundation, Chiayi, Taiwan; ^2^Rehabilitation Counseling Program, Portland State University, Portland, OR, United States; ^3^Department of Neurology, Dalin Tzu Chi Hospital, Buddhist Tzu Chi Medical Foundation, Chiayi, Taiwan; ^4^Center of Sports Medicine, Dalin Tzuchi Hospital, The Buddhist Tzuchi Medical Foundation, Chiayi, Taiwan; ^5^Department of Obstetrics and Gynecology, Dalin Tzu Chi Hospital, Buddhist Tzu Chi Medical Foundation, Chiayi, Taiwan; ^6^Division of Allergy, Immunology and Rheumatology, Dalin Tzuchi Hospital, The Buddhist Tzuchi Medical Foundation, Chiayi, Taiwan; ^7^School of Medicine, Tzu Chi University, Hualien, Taiwan; ^8^Graduate Institute of Sports Science, National Taiwan Sport University, Taoyuan, Taiwan; ^9^School of Post-Baccalaureate Chinese Medicine, Tzu Chi University, Hualien, Taiwan; ^10^Department of Occupational and Environmental Medicine, National Cheng Kung University, Tainan, Taiwan; ^11^Department of Nursing, Tzu Chi University of Science and Technology, Hualien, Taiwan; ^12^Department of Medical Research, Dalin Tzuchi Hospital, The Buddhist Tzuchi Medical Foundation, Chiayi, Taiwan

**Keywords:** low back pain, orthopedic manual therapy, pregnancy, nested case-control study, risk

## Abstract

**Background:**

Recent evidence suggests that the use of orthopedic manual therapy (OMT) may lessen the subsequent risk of low back pain (LBP), but this association has not been examined among pregnant women who are at higher risk of LBP. This study aims to determine whether the addition of OMT to conventional LBP treatment before pregnancy could decrease the subsequent risk of LBP during pregnancy.

**Methods:**

From Taiwan's National Health Insurance Research Database, we identified 68,960 women, 20–55 years of age, with first pregnancy between 2001 and 2012. We then performed a nested case-control study in which 3,846 women with newly diagnosed LBP were matched to 3,846 controls according to age and cohort entry year. Multivariate conditional logistic regression was employed to estimate the association between OMT use before pregnancy and LBP during pregnancy.

**Results:**

OMT users had a lower risk of LBP than did non-users, with an adjusted OR of 0.86 (95% CI, 0.78–0.93). Subgroup analysis showed that women with high intensity use of OMT treatment prior to pregnancy reported the lowest level of LBP during pregnancy by nearly 30%.

**Conclusion:**

The pre-pregnancy use of OMT treatment significantly decreased LBP risk during pregnancy, especially with high-intensity use. Thus, clinicians may consider recommending OMT for pregnant women to avoid possible obstetric complications during the pregnancy.

## Introduction

Low back pain (LBP) is increasingly common worldwide. Epidemiological surveys from different centuries put LBP figures, during lifetime, at between 377.5 million in 1990 and 577.0 million in 2017, translating to an increase by nearly 53% (1). LBP affects all age groups, especially women during pregnancy, affecting 7 out of 10 pregnant women (2). Extreme LBP places the affected women at increased risk of developing perinatal depression, thus compromising both maternal and pediatric health (3). Despite the high prevalence of pregnancy-related LBP and its significant impact on women's quality of life, the physiological and biomechanical processes underlying the development of pregnancy-related LBP remain unclear (4). The extra weight and body changes in pregnancy along with loosened joints and ligaments due to hormonal changes are implicated in this condition. A recent study suggests that morphometric differences in the paraspinal muscle before pregnancy may be associated with subsequent clinical symptoms, particularly LBP (5).

Acetaminophen and non-steroidal anti-inflammatory drugs are common treatments for LBP. However, such medications can cause neurological and renal problems (6). A recent cohort study in the US reports that pregnant women receiving NSAIDs experienced a 60% higher risk of miscarriage than did those who did not (7). Thus, shifting the approach to managing LBP, from treatment to early prevention, is imperative, particularly for such groups (8).

Orthopedic manual therapy (OMT), an important branch of traditional Chinese medicine, has recently become a popular form of treatment for pain (9). A randomized sham-controlled trial showed that adding OMT to conventional treatment resulted in a greater decrease in LBP and functional disability than did conventional care without OMT (8). OMT treatment was reported to have favorable therapeutic outcomes among patients with inflammatory conditions and was proposed to act *via* regulation of specific circulating cytokines and leukocytes (10, 11). In view of the growing body of evidence suggesting that abnormal inflammatory responses may contribute to a predisposition to LBP (12, 13), the use of OMT prior to or early in pregnancy might lessen the risk of having LBP in later pregnancy. Accordingly, this study aims to determine whether adding OMT to conventional treatment for LBP before pregnancy could lessen the subsequent risk of LBP during the pregnancy period.

## Methods

### Data Source and Identification of Participants

Taiwan's NHI program, launched in 1995, is a single-payer government-operated compulsory health insurance program. As of 2015, up to 99.6% of Taiwan's population was enrolled in the NHI program (14). The Longitudinal Health Insurance Database (LHID), the database for the NHI program, is made up of one million randomly beneficiaries under the NHI and contain registration profiles that cover (i) enrollees' demographic information; (ii) health insurance claims data; (iii) diagnostic codes; (iv) contracted pharmacies; and (v) medical examination information from the NHI program. Due to the use of a multistage stratified systematic sampling design, it ensures no significant deviations in the distribution of sex and age between the LHID enrollees and the general population. This study was approved by the local institutional review board and ethics committee of Buddhist Dalin Tzu Chi Hospital (No. B10803015-1).

**Figure 1:**
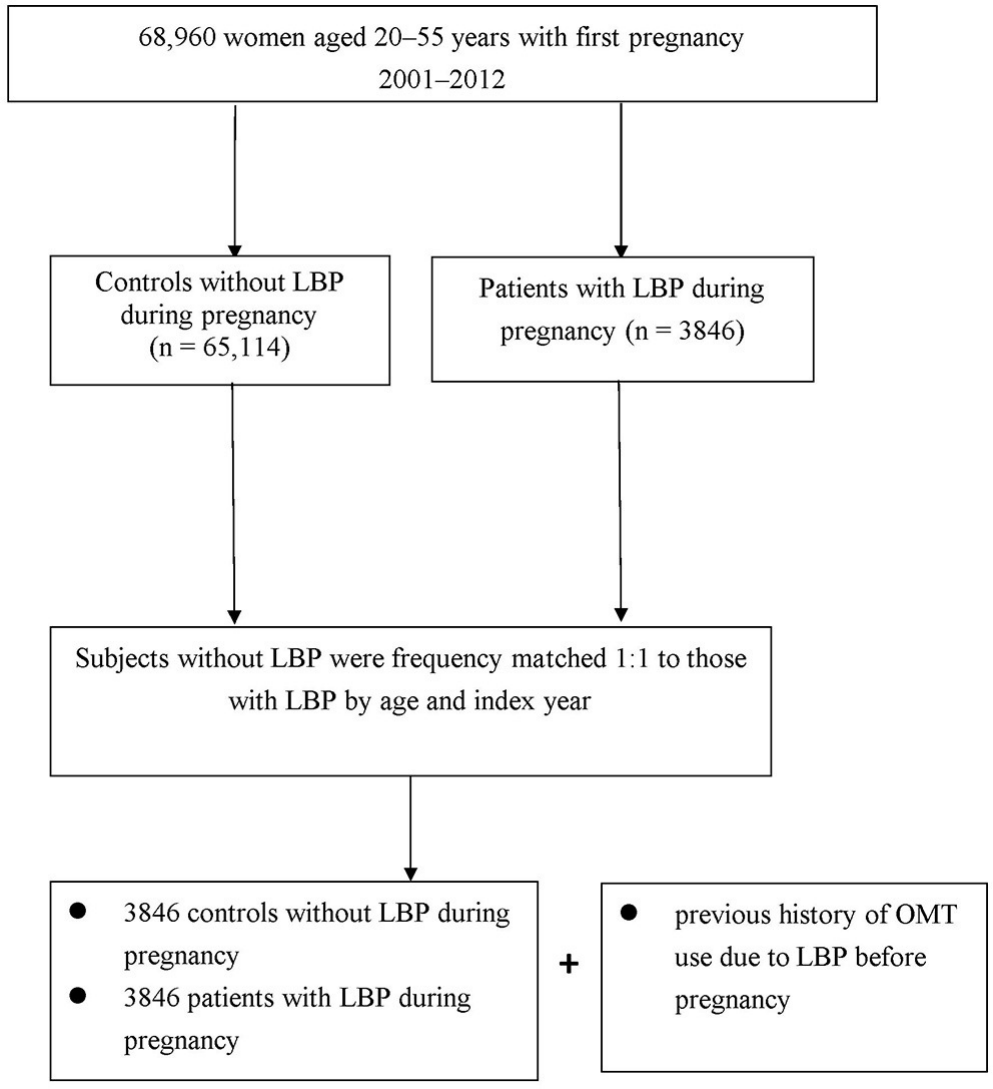
Flowchart of patient screening.

In this nested case-control study, the relevant disease designation was defined by using the International Classification of Disease, Ninth Revision, Clinical Modification (ICD-9-CM) code in the medical record diagnosis field. First, we recruited pregnant women 20–55 years of age who had a first-time diagnosis of pregnancy as V22, V23, or 640–648 in the outpatient dataset or as operation code 74 in the inpatient dataset for cesarean delivery between 2001 and 2012 (*n* = 70,638). The patients were divided into two groups according to whether they have, or have not, sought ambulatory healthcare services for LBP during their pregnancy (ICD-9-CM codes 721.3, 722.1, 724.02, 724.09, 724.2, 724.3, 724.5, 724.9) (15). To minimize the potential for disease misclassification, LBP was defined as at least three consensus diagnoses during outpatient visits, within 1 year, or hospital admission with a primary diagnosis, all with the required diagnostic code for LBP. The first LBP diagnosis was defined as the index date. The control group comprised patients randomly selected from the remaining insured pregnant women without LBP. For each patient, one matched control was selected using frequency matching for age and entry date. Each control was assigned the index date of the corresponding patient. After matching, we then analyzed the history of OMT use for LBP before pregnancy in both groups (Figure 1).

### Definition of Exposure

From the medical claims data, we defined history of OMT use as the code “C4” in records of ambulatory care visits dating from 1996, when computerized claims data from the LHID became available, until the index date. For inclusion, we required that OMT services were provided by a certified Chinese medicine physician to ensure diagnostic validity and to avoid any potential misclassifications. Under the NHI program in Taiwan, only certified Chinese medicine physicians are allowed to provide OMT therapy that contains high-velocity/low-amplitude (HVLA) and myofascial release (MFR).

### Inclusion of Potential Confounders

Potential confounders included age, monthly income (for estimating insurance payment), urbanization level of the enrollee's residential area, and previous comorbidities. Monthly income was separated into 3 classes, from lowest income (class 1) to highest income (class 3). A previous study classified urbanization into 7 levels, with a higher number indicating a more urban environment (16). In this study, we classified urbanization into 3 levels: high (metropolitan cities), medium (small cities and suburban areas), and low (rural areas). Baseline comorbidities for each woman were determined based on claims data for 1 year before index date, all of which were assessed by the established Charlson-Deyo comorbidity index (CCI) (17). It is a commonly used method of categorizing comorbid conditions of patients based on the diagnostic codes found in administrative data. Higher scores on the CCI are indicative of a more severe impact of the concomitant comorbidities.

### Statistical Modeling

All statistical analyses were carried out using SAS version 9.3 software (SAS Institute Inc., Cary, NC, USA). We used the Chi-square test and independent *t*-test to compare demographic characteristics between the patients and controls. Univariate conditional logistic regression analysis was used to estimate the crude association between each covariate and LBP during pregnancy. Multivariate conditional logistic regression analysis was used to evaluate the association between a previous history of OMT use and LBP. All multivariate models were adjusted for patient comorbidities and demographic data. Furthermore, to ensure robustness of the findings herein, we conducted a sensitivity analysis that further divided all subjects into 2 groups, according to their placement of either above and below the 50th percentile for the frequency of OMT use. This procedure allowed us to directly estimate the effect of OMT on LBP prevention. The associations are presented as odds ratio (OR) with 95% confidence interval (CI). The statistical test results were considered significant with a 2-sided *p* < 0.05.

## Results

From the original cohort, we identified 3,846 women with newly diagnosed LBP and 3,846 controls who did not experience LBP during pregnancy. The baseline characteristics of the two groups are shown in Table 1. The age distribution between them were well-matched, with a mean age of 29.60 ± 6.21 years for all enrollees (Table 1). The majority of participants had a monthly income of NTD 17,881–43,900 (81.6%) and lived in urbanized areas (58.4%). For comorbidities, the mean CCI score was 0.42 (±1.96). Collectively, no significant differences were observed between the two groups with respect to age, monthly income, location of residence, or CCI score after random matching, indicating that the two groups were comparable in baseline characteristics.

**Table 1:** Subject demographic data and comorbidities.

	**Total subjects**	**Patients**	**Controls**	***p***
	**(*n* = 7,692)**	***n* = 3,846 (%)**	***n* = 3,846 (%)**	
Age				0.90
Mean (SD)	29.60 (6.21)	29.60 (6.23)	29.59 (6.18)	
Monthly income				0.72
Low	6,278 (81.6)	3,135 (81.5)	3,143 (81.7)	
Median	1,342 (17.4)	678 (17.6)	664 (17.3)	
High	72 (0.9)	33 (0.9)	39 (1.0)	
Residential area				0.68
Urban	4,494 (58.4)	2,238 (58.2)	2,256 (58.7)	
Suburban	1,432 (18.6)	716 (18.6)	716 (18.6)	
Rural	1,766 (23.0)	892 (11.6)	874 (22.7)	
CCI				
Mean (SD)	0.42 (1.96)	0.43 (1.99)	0.42 (1.94)	0.82

*SD, standard deviation*.

We next used multivariable logistic regression models to identify the association of previous OMT use with the sequent risk of LBP during pregnancy (Table 2). Those who ever used OMT had a lower risk of LBP than those who did not (adjusted OR, 0.86; 95% CI, 0.78–0.93). Notably, high-intensity OMT was associated with a nearly 30% lower risk of LBP, which suggests a dose-dependent inverse relationship between OMT use and the risk of LBP during pregnancy (adjusted OR, 0.72; 95% CI, 0.64–0.84).

**Table 2:** The association between LBP onset and use of OMT before pregnancy.

**OMT use before pregnancy**	**Subjects [*****n*** **(%)]**				**Crude OR (95% CI)**	Adjusted OR^*^(95% CI)
	**Patients**		**Controls**			
	***n*** **=** **3,846**		***n*** **=** **3,846**			
No OMT	1,852	48.2	1,710	44.5	1	1
OMT users	1,994	51.8	2,136	55.5	0.86(0.79–0.94)	0.86 (0.78–0.93)
Low intensity	1,501	39.0	1,522	39.6	0.91(0.83–0.99)	0.91 (0.83–0.98)
High intensity	493	12.8	614	16.0	0.73 (0.65–0.85)	0.72 (0.64–0.84)

* *Model adjusted for age, residential area, monthly income, and CCI*.

## Discussion

This is the first study to our knowledge to report the OMT use prior to the pregnancy may lessen the subsequent risk of LBP during the pregnant period. We discovered that the rate of LBP throughout pregnancy was significantly lower among women who ever underwent OMT for LBP before pregnancy than among those who did not. Notably, those receiving high intensity OMT treatment had a nearly 30% lower risk of LBP than did non-OMT users. No earlier research findings have shown this relationship, rendering a comparison of results impossible. Yet, the findings obtained herein are consistent with earlier research findings and add to the growing body of knowledge on the beneficial effects of OMT for patients with chronic diseases (11, 18, 19).

While the underlying mechanisms of OMT are still not well-understood, several hypotheses have been proposed to explain the positive effect of OMT on reducing LBP. First, one study notes that use of OMT was helpful for normalizing paraspinal muscle activity and side-to-side balance, as determined by muscle functional magnetic resonance imaging (mfMRI). This treatment relieved pain and functional disability caused by lumbar muscle asymmetry, thus lessening the recurrence of LBP (20). Second, women affected by LBP are reported to have a tendency toward higher levels of pro-inflammatory cytokines such as interlukin-1 beta (IL-1), interlukin-6 (IL-6), and tumor necrosis factor-α (TNF-α), both locally and systemically (12, 13). As of now, OMT is gaining acceptance among pregnant women, with medical professionals reporting that they adopt OMT for treating chronic LBP because of its beneficial anti-inflammatory effects (8). An *in vivo* study of patients with chronic LBP showed that the inflammatory response significantly decreased after implementation of a 6-session regimen of OMT (10). Recent studies also indicated that regulation of the autonomic nervous system functions may underlie the benefits of OMT, thus resulting in the reduction of pro-inflammatory substances (11, 21). Immune cells and inflammatory responses are affected by both parasympathetic and sympathetic efferent nerves (22), with the latter implicated in the earlier stages of the inflammatory process and the former playing a decisive role in regulating innate immune responses and cytokine function in chronic process (23, 24).

While the clinical success of OMT observed in this study may support the hypothesis that OMT modulates biomechanical function and reduces LBP onset, several limitations should be considered. First, our findings were derived from analysis of retrospective claim data based on ICD-9-CM diagnostic codes. Thus, relevant cases may have been misclassified. To improve diagnostic accuracy and minimize overestimation of the prevalence of conditions of interest, we enrolled only women with a first-time diagnosis of pregnancy or LBP. LBP classification required either: (a) at least three outpatient visits reporting consistent diagnoses in 1 year or (b) at least one inpatient admission. In addition, the NHI Administration randomly reviews the records of 1 in 100 ambulatory care visits, and 1 in 20 in-patient claims to verify the accuracy of the diagnosis. Also, a certain degree of exposure misclassification is inevitable. However, because the coding approach and data set were similar, regardless of case/control status, we believe this bias to be non-differential to cause an underestimate of the association. Second, the LHID lacks information on variables such as social network relationships, family history, laboratory data, and fetal weight. Accordingly, residual confounding might occur in the observed association. Third, the records of OMT are administratively integrated and are only available in a single location, thus precluding further examination of the initial database. It is, therefore, impossible to directly identify which specific OMT was applied to each patient. As a result, data are not available to directly compare specific therapeutic effects across diverse forms of OMT. Future research to address this concern, by using clinical survey in diverse groups of individuals, is warranted. Finally, despite our balanced study design, in which several confounding factors were adequately controlled, data derived from a retrospective nested case-control study are generally of lower statistical rigor than those derived from randomized trials because of potential biases. Therefore, well-designed randomized controlled trials are needed to minimize confounding by covariates not explicitly accounted for in the present design, thus paving the way for further *in vivo* studies on the effect of OMT on patients with other chronic disorders. These limitations notwithstanding, this investigation has several strengths. A major strength of this study is that it included a large, population-based sample that is highly representative, leaving little room for selection bias. Moreover, compared with former studies, our outcomes were based on administrative codes instead of patient self-report, and our multivariate model included adjustment for several potentially significant covariates, all of which could strengthen the validity of the study findings.

## Conclusion

To our knowledge, this study is the first large-scale investigation of the effect of OMT on the risk of pregnancy-related LBP based on nationwide figures. We found that high-intensity use of OMT prior to the pregnancy indeed ameliorated LBP risk in later pregnancy by nearly 30%. These findings suggest that clinicians might consider recommending OMT to pregnant women to minimize subsequent obstetric complications and negative effects on pediatric health throughout the pregnancy period.

## Data Availability Statement

The data supporting the conclusion of this study are available from the authors, but the raw data (NHIRD) need to be obtained from the National Health Research Institute of Taiwan through an application process upon approval.

## Ethics Statement

The studies involving human participants were reviewed and approved by Institutional Review Board and Ethics Committee of Buddhist Dalin Tzu Chi Hospital. Written informed consent for participation was not required for this study in accordance with the national legislation and the institutional requirements.

## Author Contributions

Study concept and design and writing: W-CC, W-JC, HL, C-TY, M-CH, and T-YT. Acquisition of data: W-JC, M-CL, and T-YT. Data analysis: HL and T-YT. Project management: W-CC, W-JC, and T-YT. All authors contributed to the article and approved the submitted version.

## Funding

The study is based on data from the National Health Insurance Research Database, provided by the Bureau of National Health Insurance (Department of Health) and managed by the National Health Research Institutes. The interpretation and conclusions contained herein do not represent those of the Bureau of National Health Insurance, Department of Health, the National Health Research Institutes, or the study funders.

## Conflict of Interest

The authors declare that the research was conducted in the absence of any commercial or financial relationships that could be construed as a potential conflict of interest.

## Publisher's Note

All claims expressed in this article are solely those of the authors and do not necessarily represent those of their affiliated organizations, or those of the publisher, the editors and the reviewers. Any product that may be evaluated in this article, or claim that may be made by its manufacturer, is not guaranteed or endorsed by the publisher.
